# Placental Site Trophoblastic Tumor Acquires Immune Functions by Incorporating Host Maternal Genes

**DOI:** 10.1002/advs.76071

**Published:** 2026-06-23

**Authors:** Kyosuke Kagami, Masanori Ono, Yasunari Mizumoto, Tatsuhito Kanda, Takashi Iizuka, Takiko Daikoku, Shin‐ichi Horike, Akira Hattori, Akihito Horie, Sachiko Minamiguchi, Tomoko Fujiwara, Kazuyoshi Hosomichi, Atsushi Tajima, Hirokazu Usui, Kaoru Abiko, Hiroshi Fujiwara

**Affiliations:** ^1^ Department of Obstetrics and Gynecology Graduate School of Medical Sciences Kanazawa University Kanazawa Japan; ^2^ Department of Obstetrics and Gynecology Tokyo Medical University Shinjuku Tokyo Japan; ^3^ Department of Obstetrics and Gynecology Keio University School of Medicine Shinjuku Tokyo Japan; ^4^ Department of Obstetrics and Gynecology Ishikawa Prefectural Central Hospital Kanazawa Japan; ^5^ Division of Animal Disease Model Research Center for Experimental Modeling of Human Disease Kanazawa University Kanazawa Japan; ^6^ Division of Integrated Omics Research Research Center for Experimental Modeling of Human Disease Kanazawa University Kanazawa Ishikawa Japan; ^7^ Department of System Chemotherapy and Molecular Sciences Kyoto University Graduate School of Pharmaceutical Sciences Kyoto Japan; ^8^ Department of Gynecology and Obstetrics Kyoto University Graduate School of Medicine Kyoto Japan; ^9^ Department of Obstetrics and Gynecology Medical Research Institute KITANO HOSPITAL Osaka Japan; ^10^ Department of Diagnostic Pathology Fujita Health University School of Medicine Aichi Japan; ^11^ Department of Human Life Environments Kyoto Notre Dame University Kyoto Japan; ^12^ Laboratory of Computational Genomics School of Life Science Tokyo University of Pharmacy and Life Sciences Hachioji Tokyo Japan; ^13^ Department of Bioinformatics and Genomics Graduate School of Advanced Preventive Medical Sciences Kanazawa University Kanazawa Japan; ^14^ Department of Obstetrics and Gynecology Reproductive Medicine Chiba University Graduate School of Medicine Chiba Japan; ^15^ Ochi Yume Clinic Nagoya Nagoya Japan; ^16^ School of Veterinary Medicine Azabu University Sagamihara Japan

**Keywords:** cell fusion, gene incorporation, gene rearrangement, immunoglobulin, PD‐1, placental site trophoblastic tumor

## Abstract

Although it was proposed that cell fusion of cancer cells with leukocytes creates mobile hybrids with a metastatic phenotype, it has been difficult to genetically confirm cell fusion events in human cancer in vivo. Here, we experienced 4 cases of placental site trophoblastic tumor (PSTT) that produced immunoglobulin (Ig). Three cases showed recurrence and responded well to pembrolizumab therapy. Among them, we could analyze temporal changes in the genetic profiles on one case of daughter‐derived PSTT, which relapsed after pembrolizumab therapy. In this case, we found that PSTT incorporated the exogenous genes from host maternal cells. The rearrangement patterns of Ig genes and protein expressions sequentially increased. By analyzing single‐nucleotide variants, PSTT incorporated daughter‐non‐inherited maternal alleles (DNIMA), including the Ig lambda and HLA‐DQA2 loci. Protein expressions of TLR10 and SIGLEC10 increased during tumor progression concomitantly with DNIMA incorporation. DNIMA mapping indicates the incorporation of exogenous maternal genes was widely distributed through the whole chromosomes, suggesting the involvement of cell fusion in gene transfer mechanisms. These findings indicate that PSTT sequentially incorporated exogenous genes from maternal cells to express immune‐related molecules and suggest that cancer cells acquired B cell‐related functions, including Ig production by cell fusion with host immune cells.

## Graphical abstract

PSTT cells, through cell fusion with B cells, incorporate abundant non‐inherited maternal genes that are detectable by DNIMA. These hybrid cells acquire immunotherapy‐resistant genetic changes and increase the expression of B cell‐derived immune‐related molecules such as Ig, HLA, LILRB, SIGLEC10, and so on, creating an immunotolerant environment around the tumors.

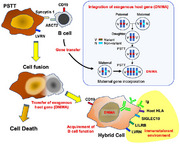

**Abbreviations**
BMDC: bone marrow‐derived cells
CT: computed tomography
DNIMA: daughter‐non‐inherited maternal allele
EMT: epithelial‐mesenchymal transition
ERV: endogenous retrovirus
FFPE: formalin‐fixed paraffin‐embedded
hCG: human chorionic gonadotropin
Ig: immunoglobulin
LILRB: leukocyte immunoglobulin‐like receptor subfamily B member
LINE: long interspersed nuclear elements
LTR: long terminal repeat
LVRN: laeverin
Mel‐CAM: melanoma cell adhesion molecule
Mhon/Dhe‐VS: maternal‐homo‐non‐variant and daughter‐hetero‐variant sequence
MSI: microsatellite instability
PBMC: peripheral blood mononuclear cell
PD‐1: programmed cell death protein 1
PSTT: placental site trophoblastic tumor
Siglecs: sialic acid‐binding Ig‐type lectins
SINE: hort interspersed nuclear elements
SNV: single nucleotide variant
STR: short tandem repeat
SVA: SINE‐VNTR‐Alu
THCs: tumor hybrid cells

## 1 Introduction

Ever since a German pathologist, Otto Aichel, proposed that cell fusion of cancer cells with leukocytes creates mobile hybrids with a metastatic phenotype in 1911, evidence to support this concept has been reported using in vitro and animal experiments [1, 2]. Currently, based on accumulating clinical evidence, the fusion of cancer cells with myeloid cells has attracted attention [3]. By investigating female cancer patients who had previously received a sex‐mismatched bone marrow transplant, Y chromosome‐positive hybrid cancer cells have been detected [4]. Furthermore, the immunohistochemical study showed the presence of cytokeratin‐positive hybrid cancer cells with Y chromosome–containing nuclei [5]. In addition, CD45/EpCAM double‐positive circulating cells with Y chromosome‐positive nuclei, which are suggested to be derived from cell fusion between a peripheral mononuclear blood cell and an epithelial tumor cell, were observed and shown to be related with disease stage and predict overall survival [5].

The phenomenon of cell fusion between lymphocytes and non‐lymphocytes was previously reported [6]. It was also demonstrated that tetraploid hybrids produced by cell fusion between murine bone marrow‐derived cells and hepatocytes, or human lymphoma cells and endothelial cells, undergo reduction division, resulting in diploid daughter cells in vivo [6, 7, 8]. The cell fusion of cancer cells with myeloid cells cell was reported to generate hybrid cells, so‐called tumor hybrid cells (THCs). The fate of cancer hybrid cells is highly variable, with the heterokaryon‐to‐synkaryon transition and ploidy reduction, leading to aneuploidy, genomic instability, DNA damage, or micronuclei formation. Thus, the survival probability of tumor hybrids is considered rather rare [9]. On the other hand, the survived cancer hybrid cells are considered to enhance cancer cell properties such as migratory ability, angiogenesis, immune evasion, colonization, and metastasis. Cancer hybrid cells were also proposed to change the therapeutic and apoptotic responses of cancer stem cells [9]. Consequently, this process has been proposed to lead to tumor progression, drug resistance, and cancer recurrence [3]. Among bone marrow‐derived cells (BMDC), several experiments suggest that the monocyte/macrophage lineage cells are the main counterparts for hybrid cells with cancer cells to acquire cancer cell properties of epithelial‐mesenchymal transition (EMT) and metastasis [10]. However, it has been difficult to sequentially observe cell fusion events in human cancer in vivo because the genetic origins of DNA between tumor cells and bone marrow‐derived cells cannot be easily distinguished [11].

Placental site trophoblastic tumor (PSTT) is a very rare malignant trophoblastic disease. It originates from embryo‐derived extravillous trophoblasts (EVT) [12] and has allogeneic paternal antigens. During the early stage of placental formation, EVT proliferates in the anchoring villi, invades the maternal endometrium, and reconstructs maternal spiral arteries without rejection from the maternal immune system. Here, we encountered four cases of PSTT that produced Ig proteins. Among them, we were able to analyze temporal changes in the genetic profiles of one case, which was derived from the daughter and showed repeated local recurrences and transiently responded well to pembrolizumab therapy. Intriguingly, the rearrangement patterns of Ig genes in tumors were increased during tumor progression. In addition, the immunohistochemical expression of immunomodulating molecule, leukocyte immunoglobulin‐like receptor subfamily B members (LILRB), was increased with tumor recurrence. Currently, the acquisition of immuno‐resistance against immune checkpoint blockade therapy has become a major concern in cancer therapy [13, 14]. Since EVT has a high capacity for cell fusion [15], we assumed that PSTT incorporated maternal genes of immune cells, including B lymphocyte‐lineage cells, by cell fusion to acquire Ig producing and enhance immune‐tolerant ability.

In this study, to validate the above hypothesis, we further performed single‐cell gene analysis of PSTT, analyzed single‐nucleotide variant sites (SNVs) that can distinguish embryo‐non‐inherited maternal alleles from embryo‐inherited ones, created chromosomal mapping of incorporated SNVs, and evaluated the presence of genetically hybrid tumor cells that had exogenously incorporated maternal cell genes.

## 2 Results

### 2.1 Clinical Course of PSTT

**FIGURE 1:**
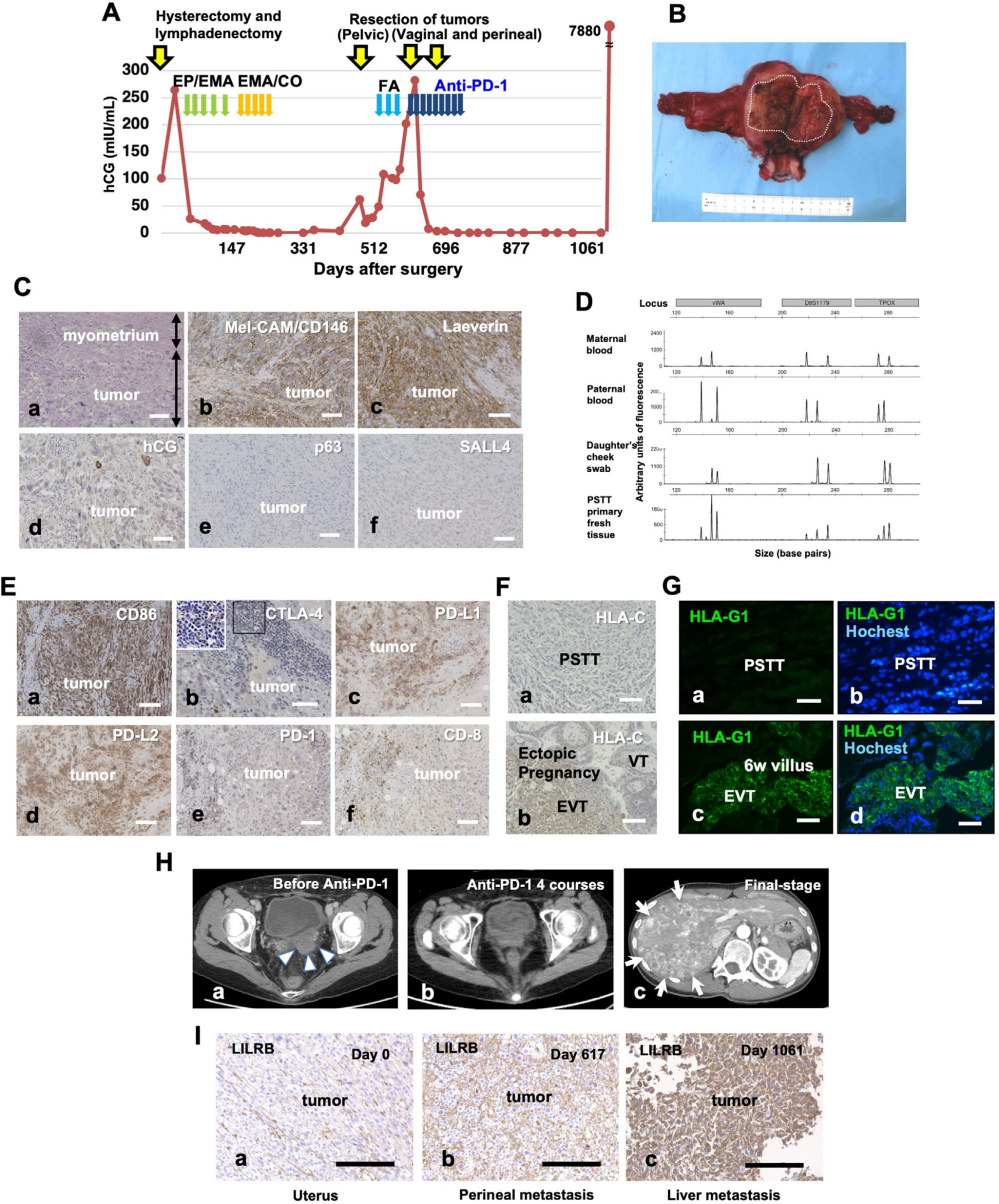
Clinical data and expression of immune‐related molecules (A) The clinical course of PSTT (patient #1). (B) The removed uterus showed the primary lesion of PSTT (white dotted area). (C) Histologically, hematoxylin and eosin staining confirmed myometrial invasion by EVT‐like polygonal malignant cells (a). As characteristics of PSTT, these tumor cells were strongly positive for Mel‐CAM (b) and laeverin (c), partially positive for hCG (d), but negative for p63 (e) and SALL4 (f). (D) Selected short tandem repeat analysis revealed that it was the gestational PSTT that was derived from the daughter. (E) This tumor expressed CD86 (a), PD‐L1 (c), and PD‐L2 (d), and showed the infiltration of CTLA‐4‐positive‐, CD8‐positive‐, and PD‐1‐positive T cells into PSTT (b, e, and f). Both HLA‐C (Fa) and HLA‐G1 (Ga and b) were negative for PSTT. As reported previously, villus trophoblasts (VT), including syncytiotrophoblasts and cytotrophoblasts, were negative, whereas EVT was positive for HLA‐C (Fb) and HLA‐G1 (Gc and d). (H) CT images during anti‐PD‐1 treatment. (I) During tumor progression, LILRB expression on tumor cells was gradually increased until the final stage. Scale bars: 100 µm.

Clinical outcome of 4 cases of PSTT was summarized in Table 1. Patient #1 (aged 32 years), para 3/0/0/3, with a history of 2‐year amenorrhea after the third delivery, received a total hysterectomy with bilateral salpingo‐oophorectomy and regional lymphadenectomy under the diagnosis of PSTT (Figure 1A,B). This tumor was histologically composed of EVT‐like malignant cells with polyhedral and eosinophilic cytoplasm, with a high mitotic rate and myometrial infiltration (Figure 1Ca). EVT markers, melanoma cell adhesion molecule (Mel‐CAM) and laeverin (LVRN) [16], were diffusely positive and hCG was focally positive, whereas p63, an epithelioid trophoblastic tumor marker, and SALL4, a choriocarcinoma marker, were negative (Figure 1Cb–f). STR polymorphism analysis revealed that this tumor had genes of both maternal and paternal origins, and the patterns were identical to those of the daughter (Figure 1D), confirming that this case was gestational PSTT. After sequential chemotherapies, she repeatedly underwent resection of recurrent tumors during salvage chemotherapy (Figure 1A). Genome mutation analysis showed no significant result, and microsatellite instability was low. This tumor expressed CD86, PD‐L1, and PD‐L2 (Figure 1Ea,c,d), and showed the infiltration of CTLA‐4‐positive‐, CD8‐positive‐, and PD‐1‐positive T cells into PSTT (Figure 1 Eb, e, and f), whereas HLA‐C and HLA‐G1 were negative (Figure 1F,G). Therefore, we selected pembrolizumab therapy [17]. After 9 courses, the patient was in complete remission with undetectable serum hCG levels for more than one year. However, she suddenly developed recurrence in the pancreas and liver. The progression was very rapid, and the patient died of multiple organ failure (Figure 1A,H). During tumor progression, immunohistological expression of LILRB molecules on tumor cells sequentially increased until the final stage (Figure 1I).

**TABLE 1:** Patients' information retrieved from the medical records.

Case	#1	#2	#3	#4
Age (years)	32	32	39	45
Stage and extrauterine lesion	Stage IV para‐aortic lymph node	Stage I Re	Stage I	Stage I
Metastatic lesion after recurrence	Liver, pancreas	Lung, pancreas	N/A	Multiple metastases
Therapy	H/Ch/T/I	C/Ch/I	H	H/Ch/I
Initial response to I	PR	PR	N/A	PR
Current status/follow‐up periods (months)	DOD/36	LWD/57	NED/92	LWD/29
Serum hCG at initial therapy (mU/mL)	264	373	1872	0.2
Gravidity/parity	3G3P	2G1P	3G2P	3G3P
Latest pregnancy	TD	SA	TD	TD
Pregnancy interval (months)	27	1	6	22

Re, recurrent; H, hysterectomy; T, tumorectoy; C, curettage; Ch, chemotherapy; I, immune checkpoint blockade therapy; PR, partial response; N/A, Not Applicable; DOD, died of disease; LWD, living with disease; NED, no evidence of disease; hCG, human chorionic gonadotropin; TD, term delivery; SA, spontaneous abortion.

### 2.2 PSTT Included Rearranged Ig Genes

**FIGURE 2:**
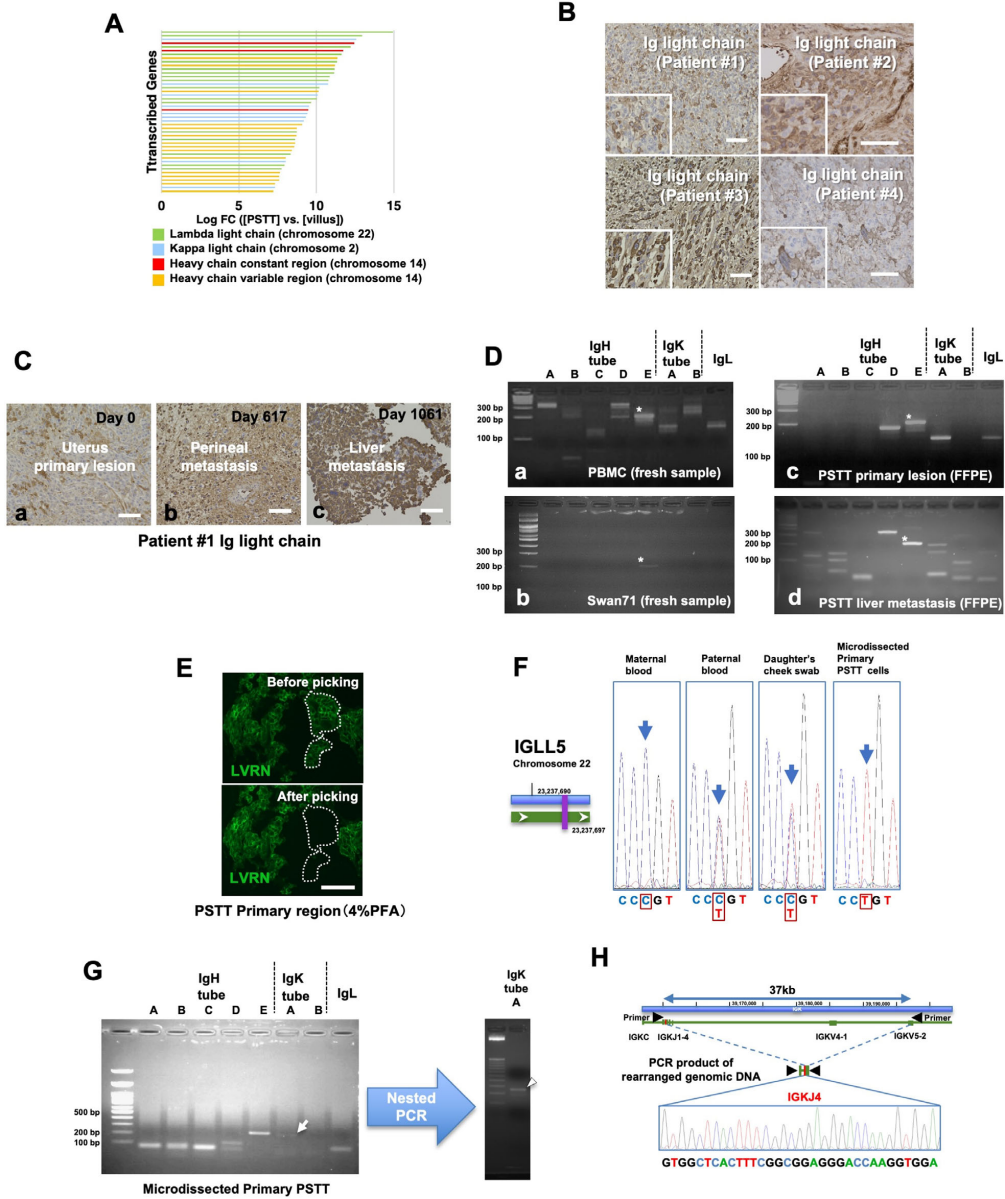
PSTT included rearranged Ig genes (A) Selected data of Ig‐associated molecules within the top 100 upregulated genes by microarray gene expression analyses between PSTT and 6‐week gestational villus tissue. The expressions of various Ig related genes were upregulated. (B) An immunoreactive Ig light chain was detected in PSTT cells in patients #1, 2, 3, and 4. (C) The expression of Ig light chain in patient #1 was promoted during tumor progression toward the final stage. (D) By multiplex PCR, polyclonal rearrangement patterns of Ig genes were observed in primary PSTT lesions and increased during tumor progression toward the final stage. PBMC (positive control, post‐rearrangement) and Swam71 cells (negative control, no rearrangement). Asterisks in tube E show the germline DH‐JH (diversity gene to joining gene segment of heavy chain) band. (E) Dozens of LVRN‐positive PSTT cells in primary lesions on stained slides were picked by microdissection under a microscope. (F) The LOH region of the IGLL5 gene locus showed only paternal SNVs, indicating no contamination of the patient's own cells. (G) Using these picked cells, we verified the rearrangement on the band of IgK tube A (arrow), which was amplified by nested PCR (arrowhead). (H) The deciphered sequences of the band showed the rearranged IGKJ4 region. Scale bars: 100 µm.

In this tumor, microarray analysis showed that the expressions of various Ig genes, including both gamma and kappa light chains, and had multiple variable V and C regions, and IgG1 heavy chain‐constant regions, were upregulated as compared with 6‐week gestational normal villus tissues, suggesting that they produced polyclonal Ig that underwent class‐switch recombination (Figure 2A). The high expression of Ig proteins was also observed in the other three cases (patients #2 – #4, Figure 2B and Table 1). The expression of Ig proteins in patient #1 was promoted during tumor progression toward the final stage (Figure 2C). By multiplex PCR, healthy adult female PBMC (positive control) exhibited PCR products showing Ig gene recombination (Figure 2 Da), whereas no recombination band was observed in Swan71 cells (negative control: a cell line derived from human normal extravillous trophoblast) (Figure 2Db). Then, we dissected the PSTT cell regions that were negative for CD19 (B‐cell marker) and CD138 (plasma cell marker) in case #1 (Figure S1). Using these formalin‐fixed paraffin‐embedded (FFPE) samples, polyclonal rearrangement patterns of Ig genes were observed in primary PSTT lesions (Figure 2Dc). The number of rearrangement patterns increased during tumor progression toward the final stage (Figure 2Dd). Although long bands were not detected, probably due to degradation and fragmentation of DNA, this suggests that PSTT sequentially incorporated exogeneous Ig genes from maternal immune cells since Ig gene rearrangement cannot be repeated.

Furthermore, to avoid contamination of the patient's somatic cells, immunostaining of LVRN, an EVT‐specific cell surface marker [16], was performed using OCT‐embedded non‐fixed fresh frozen sections. LVRN‐stained PSTT cells in the primary lesion were micro‐dissected using UniPick (Nepa Gene Co., Chiba, Japan) under a microscope and dozens of cells were collected (Figure 2E). To further confirm the purity of PSTT cells, we screened the site of loss of heterozygosity (LOH) in PSTT genes that consisted of only paternally derived genes. First, we extracted the sites of SNVs from the data of exome sequencing and then analyzed each sequence of SNV sites of DNAs derived from the family and the micro‐dissected PSTT cells by the Sanger method. The results showed that only paternal SNVs representing LOH were present at the IGLL5 locus in PSTT (Figure 2F), indicating that there was no contamination of the patient's (maternal) somatic cells in the micro‐dissected PSTT cells. Using this sample, we detected a specific band of IgK tube A (Figure 2G, arrowhead), the sequence of which was identical to the rearranged IGKJ4 region (Figure 2H). These findings confirmed that PSTT included rearranged Ig genes.

**FIGURE 3:**
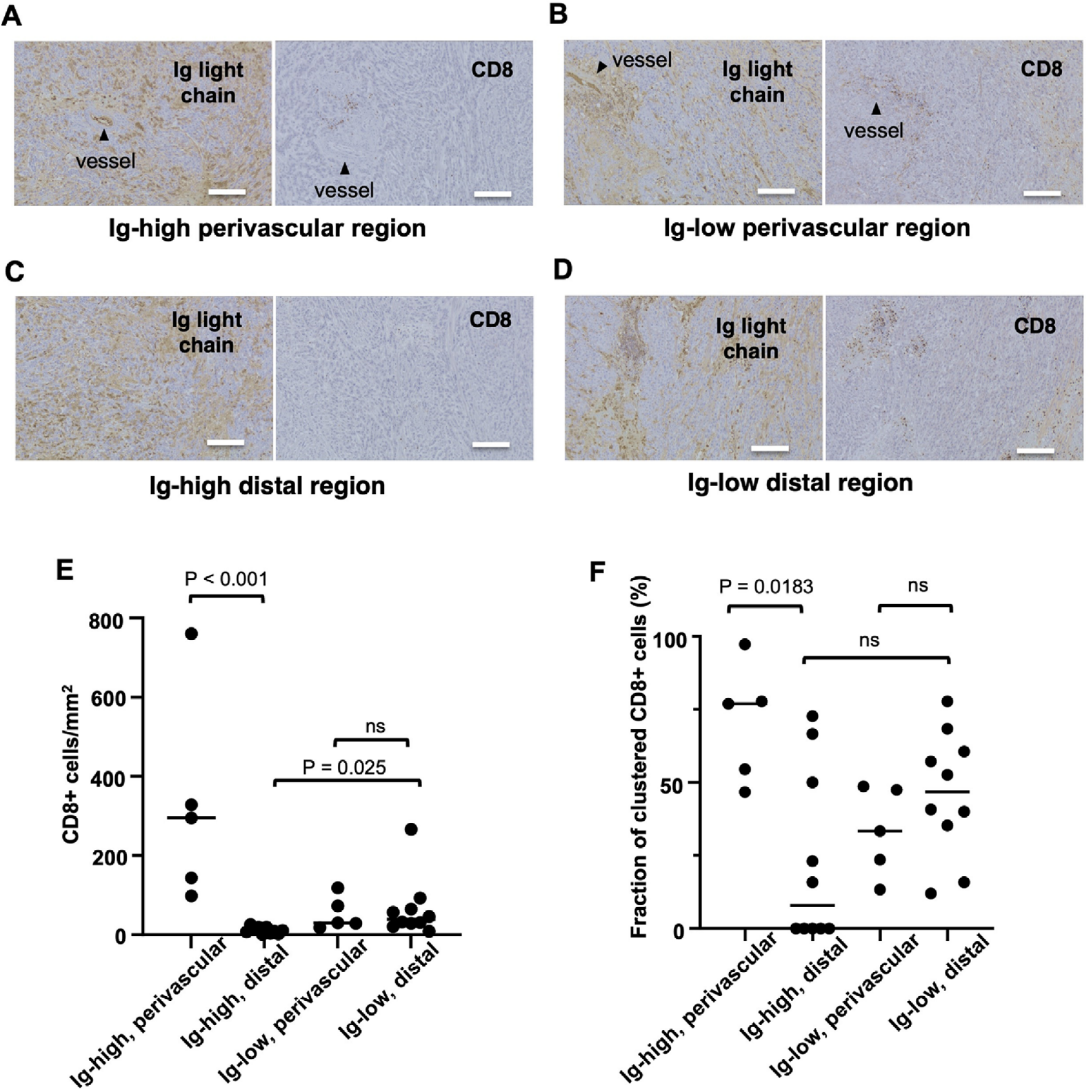
Spatial distribution and clustering of CD8‐positive T cells in Ig‐high and Ig‐low regions of PSTT (A‐B) Representative immunohistochemical images of Ig light chain (left) and CD8 (right) staining in Ig‐high (A) and Ig‐low (B) perivascular regions. In Ig‐high regions, CD8‐positive cells are predominantly localized around blood vessels with limited infiltration into the tumor parenchyma. In contrast, Ig‐low regions show both perivascular clustering and scattered infiltration. Arrowheads indicate blood vessels. Scale bars 200 µm. (C‐D) Representative images of Ig‐high (C) and Ig‐low (D) distal regions. In Ig‐high distal regions, CD8‐positive cells are largely absent. In contrast, Ig‐low distal regions exhibit both clustered and scattered CD8‐positive cells within the tumor stroma. Scale bars 200 µm. (E) Quantification of CD8‐positive cell density (cells/mm^2^) across ROIs. In Ig‐high regions, CD8‐positive cell density was significantly higher in perivascular regions compared to distal regions (*p* < 0.001). In Ig‐low regions, CD8‐positive cell density in distal regions was significantly higher than that in Ig‐high regions (*p* = 0.025). (F) Fraction of clustered CD8‐positive cells in each ROI. In Ig‐high regions, the clustered fraction was significantly higher in perivascular regions compared to distal regions (*p* = 0.0183).

Then, to access the influence of Ig expression in PSTT on the cytotoxic T lymphocytes, we examined the differences in immunohistological distribution of CD8‐positive T cells between in Ig‐high and Ig‐low expression regions of primary PSTT (Figure 3A–D). In Ig‐high regions, CD8‐positive cells were predominantly localized only around blood vessels (Figure 3A) with very limited infiltration into the distal tumor parenchyma (Figure 3C), whereas CD8‐positive cells in Ig‐low regions were observed around both the perivascular (Figure 3B) and distal sites (Figure 3D) with no significant difference. The distribution number of CD8‐positive cells in the distal sites of tumor parenchyma was significantly lower in Ig‐high regions than that in Ig‐low regions (Figure 3E), while the ratio of clustered CD8‐positive cells in Ig‐high regions was also significantly reduced from the perivascular to distal sites of tumor parenchyma (Figure 3F), suggesting the suppressing effects of Ig expression in PSTT on the function of cytotoxic T lymphocytes.

### 2.3 PSTT Incorporated Exogenous Maternal Genes

**FIGURE 4:**
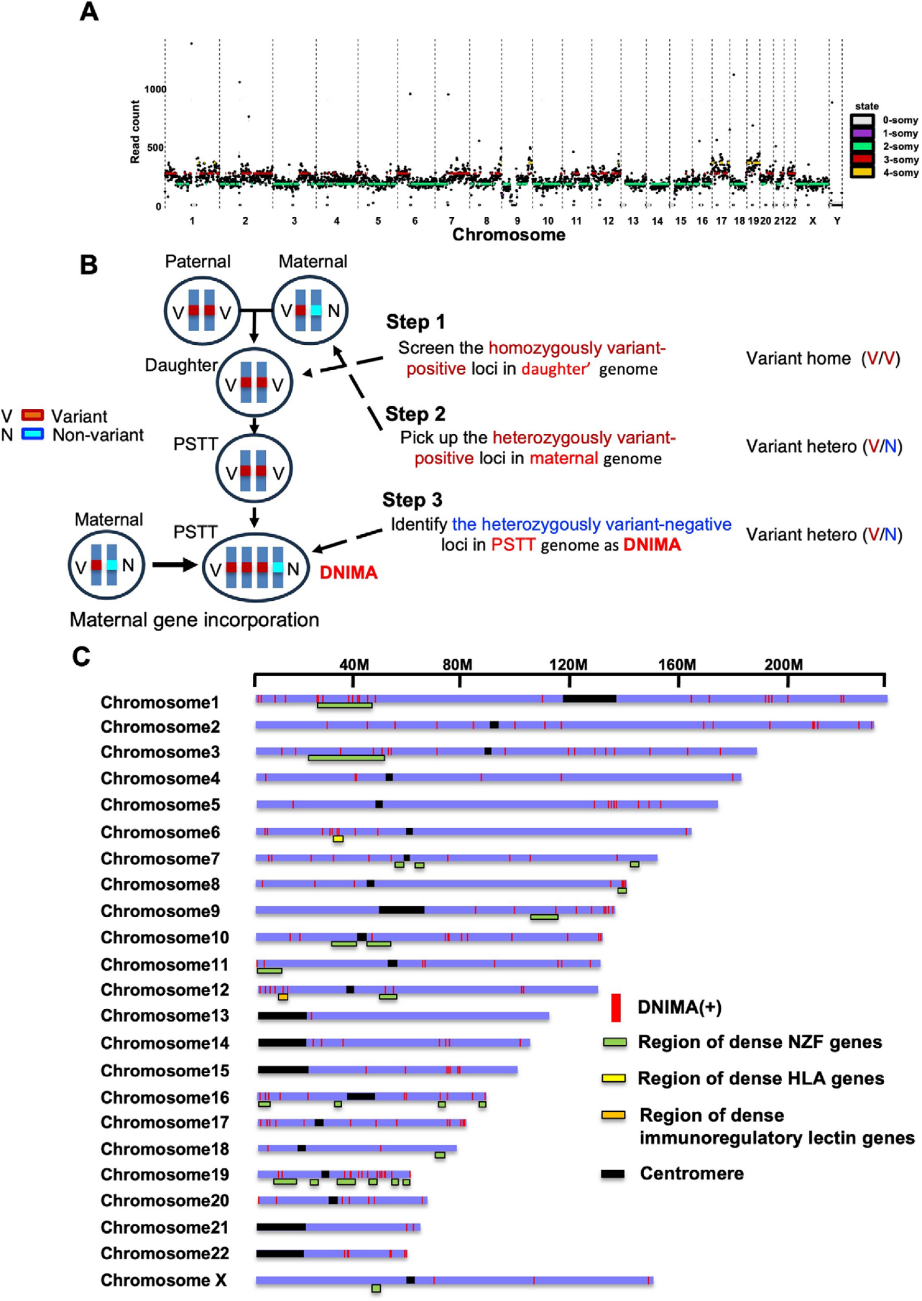
DNIMAs detected by single‐cell analysis of the whole genome sequence (A) Single‐cell whole genome sequencing‐based copy number analysis. There were many polyploidy regions. (B) Strategy to detect DNIMAs. (C) Chromosomal mapping of detected DNIMAs. There were numerous DNIMA‐incorporated sites throughout the genes.

Next, we picked up a single LVRN‐stained PSTT cell (Figure S2A). After verifying no contamination of maternal cells by analyzing LOH locus of IGLL5 (Figure S2B), we performed the whole genome sequence analysis. The results showed that there was a considerable number of polyploidy regions in each chromosome (Figure 4A). We identified SNV sites that can be used to distinguish daughter‐non‐inherited maternal alleles (DNIMA) from daughter‐inherited maternal alleles. Specifically, based on the whole genome sequencing data from the maternal blood cell and daughter’ swab samples, we first screened the homozygously positive loci for reference variants throughout the daughter's whole genome sequence, then picked up the heterozygously positive loci from the same sites in the maternal whole genome sequence. Among them, we identified the heterozygously SNV‐positive loci in the whole genome sequence determined from a single LVRN‐stained PSTT cell, defining the SNV‐negative alle as DNIMA that is exogenously incorporated from maternal cells (Figure 4B). A mapping of DNIMA showed that there were numerous DNIMA‐incorporated sites throughout the whole chromosomes (Figure 4C).

**FIGURE 5:**
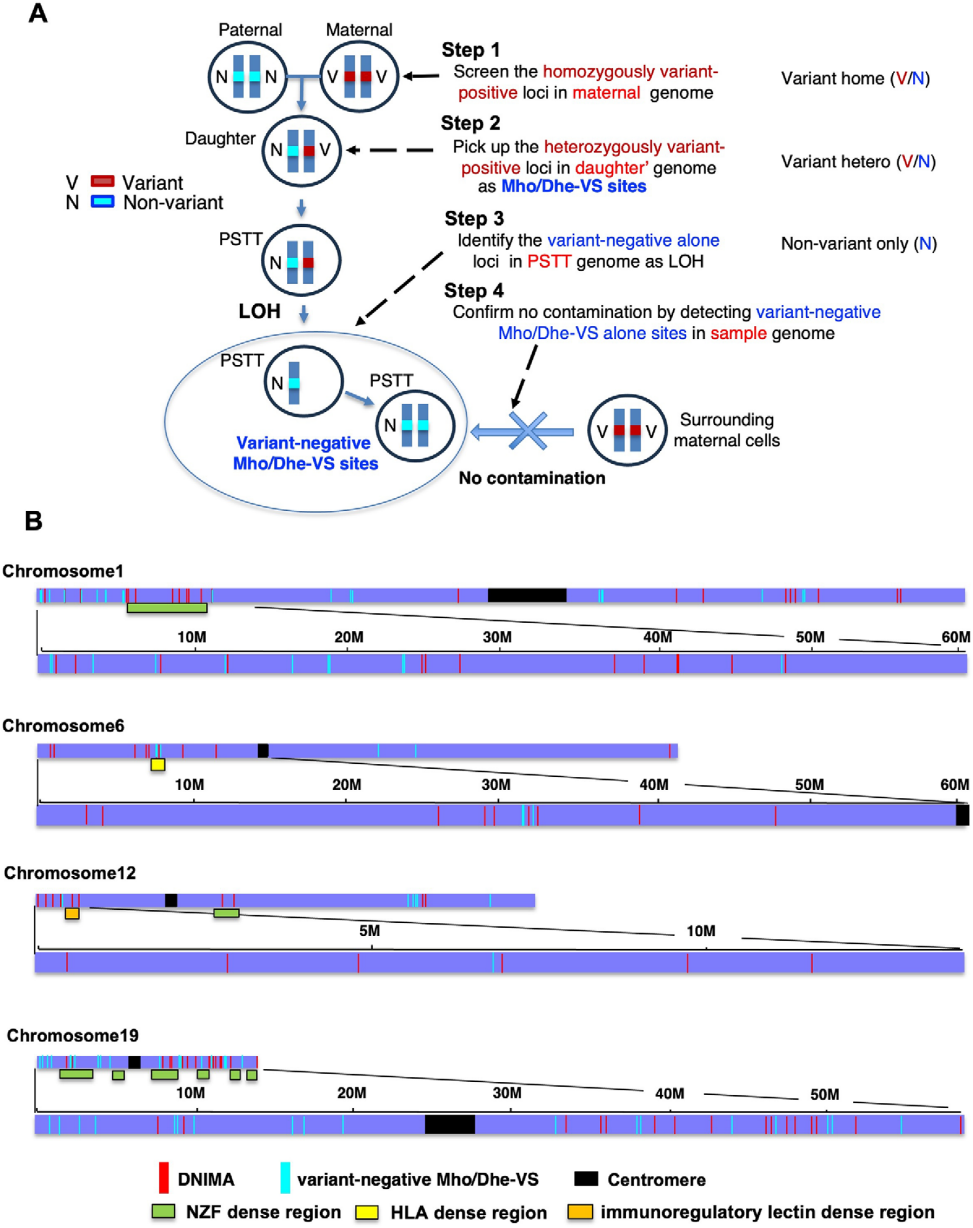
Variant‐negative Mho/Dhe‐VS alone sites detected by the whole genome sequence (A) Strategy to detect variant‐negative Mho/Dhe‐VS alone sites. (B) The chromosomal map showed that paternal variant‐negative Mhon/Dhe‐VS alone sites were distributed widely throughout chromosomes 1, 6, 12, and 19. These sites included DNIMA‐positive regions, indicating that DNIMA‐positive alleles were not derived from maternal cell contamination.

To further confirm the absence of maternal cell contamination, based on the maternal and daughter's whole genome sequencing data, we searched for loci where the mother was homozygously positive for reference variants while the daughter was heterozygously positive for the same variant, referred to as maternal‐homozygous/daughter‐heterozygous variant sequence (Mho/Dhe‐VS) sites. Among these Mho/Dhe‐VS loci, we further identified the sites where the paternal‐derived (non‐maternal) variant‐negative allele alone was positive using the PSTT single‐cell whole genome sequencing data. These variant‐negative Mho/Dhe‐VS alone sites correspond to loci of LOH with specific loss of maternal allele, which can be used to verify the absence of maternal cell contamination (Figure 5A). The chromosomal map of variant‐negative Mho/Dhe‐VS alone sites showed that these loci were distributed widely throughout the chromosome. These sites included DNIMA‐positive regions, indicating that DNIMA‐positive alleles were not derived from maternal cell contamination (Figure 5B).

Then, we examined the correlation of DNIMA‐positive sites with transposon hotspots such as long interspersed nuclear elements (LINE) [18], short interspersed nuclear elements (SINE) [19], long terminal repeat (LTR) retrotransposons [20], endogenous retrovirus (ERV) [21], and SINE‐VNTR‐Alu (SVA) retrotransposon [22]. To map the retrotransposons onto the genome, we calculated the occupancy rate of the above retrotransposons for each 1 Mb section from the whole genome data and defined the highest density sites of the top 5% as a region of dense retrotransposon. This mapping showed no significant relationship between DNIMA‐positive sites and transposon hotspots (Figure S3). As another fusion hotspot, we also examined the AT‐rich sequence sites [23, 24] and found no significant correlation with DNIMA‐positive sites (Figure S4).

### 2.4 PSTT Sequentially Incorporated Immune‐Related Functional Genes From Maternal Cells

**FIGURE 6:**
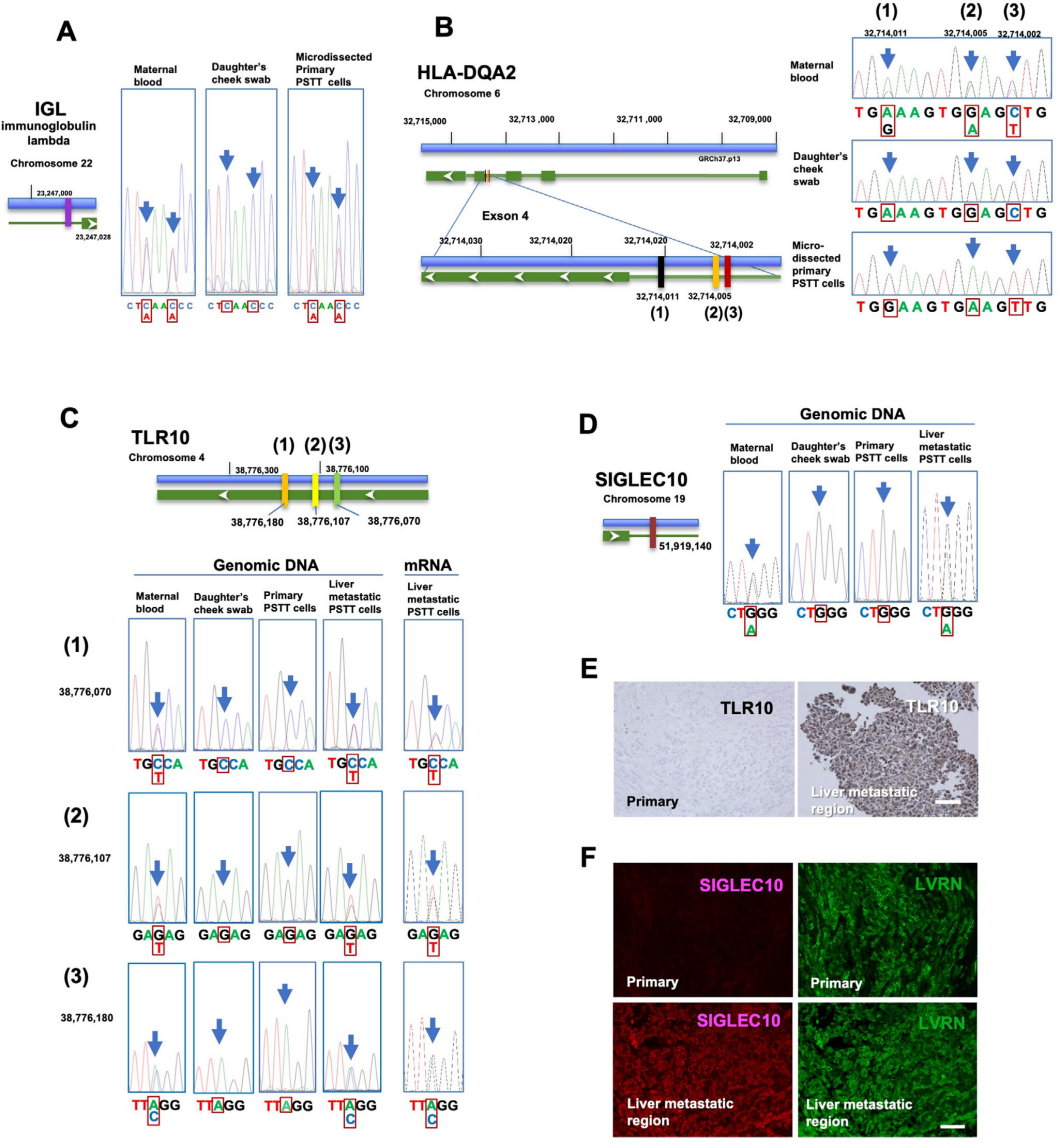
PSTT sequentially incorporated immune‐related functional genes from maternal cells. Based on the single cell‐derived mapping of DNIMA in Figure 3, we detected DNIMA in the coding region of the intronic immunoglobulin lambda locus IGL (A) and HLA‐DQA2 (B), exonic TLR10 (C), and intronic SIGLEC10 (D) in PSTT cells that were micro‐dissected from metastatic liver lesions at the final stage. Note that mRNA derived from DNIMA in TLR10 was also detected (C, right‐most panel). (E and F) Immunohistochemical staining showed strong expressions of TLR10 (E) and SIFLEC10 (F) proteins in the metastatic liver lesions, whereas they were not expressed in the primary lesions. Scale bars: 100 µm.

Based on the DNIMA map, we searched for DNIMA sites in immunoglobulin loci. Using the LVRN‐positive non‐contaminated tumor cells as described above (Figure 2E,F), we verified each sequence of DNIMA by the Sanger method and detected the DNIMA site in the intron of the Ig lambda locus (Figure 6A). This indicated the ability that PSTT produced Ig derived from exogenously incorporated maternal genes. Similarly, we verified the presence of DNIMA in the intronic region of HLA‐DQA2 (Figure 6B). These findings indicate that PSTT incorporated non‐hereditary maternal MHC class II genes. On the other hand, we found DNIMAs in the exon region of TLR10, an immuno‐respondable receptor, and in the intron region of SIGLEC10, an immuno‐suppressive receptor [25]. These DNIMAs were negative in PSTT cells of primary lesions, whereas they became positive in those from metastatic liver lesions at the final stage (Figure 6C,D). In the single‐cell‐derived mRNAs, exonic DNIMA of TLR10 was also detected (Figure 6C, rightmost column). The purity of PSTT was verified by IGLL5 loss of heterozygosity. Furthermore, immunohistochemical staining showed high‐level expressions of TLR10 and SIGLEC10 proteins in the metastatic liver lesions, whereas they were not expressed in the primary lesions (Figure 6E,F). These findings indicate that PSTT sequentially incorporated immune‐related functional genes from maternal cells and increased their protein expression as the disease progressed.

### 2.5 PSTT Expressed Cell‐Fusion Proteins

**FIGURE 7:**
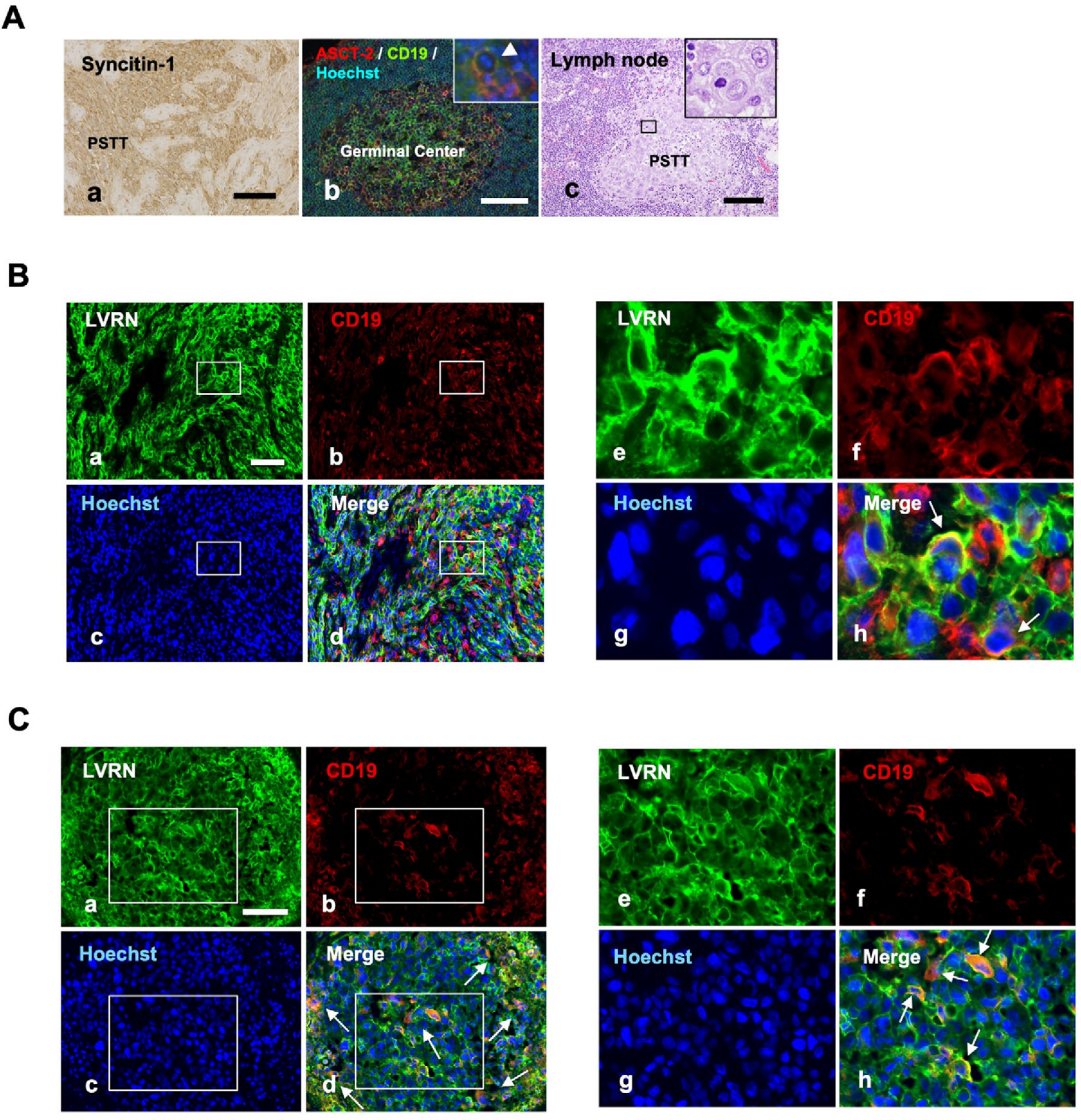
CD19‐positive B cells co‐expressed ASCT2 in the lymph node, and LVRN‐positive PSTT co‐expressed CD19 in the primary lesions (A) Syncytin‐1expression in PSTT cells and ASCT2 expression on CD19‐positive B cells in the lymph node. Syncytin‐1 was expressed on tumor cells (a). In lymph nodes, CD19‐positive B cells in the germinal center expressed their receptor, ASCT2 (b). In the germinal center of the metastatic lymph nodes, the close contact of immune cells with PSTT cells was observed (c). Scale bars: 100 µm. (B and C) Co‐expression of CD19 and LVRN in PSTT cells in the primary muscular (B) and intravascular (C) invading sites. Figures (e‐h) are the magnified images of the squared areas in figures (a‐d). (B) In some myometrial invading lesions, LVRN/CD19 double‐positive PSTT cells were detected (d and h, arrows). (C) Abundant LVRN/CD19 double‐positive PSTT cells were sporadically observed in the vascular invading sites (d and h, arrows). Scale bars: 100 µm.

Syncytin‐1 is a cell‐fusion protein derived from endogenous retrovirus envelope genes [26]. In PSTT, syncytin‐1 was expressed on tumor cells (Figure 7Aa), whereas the expression of its receptor, sodium‐dependent neutral amino acid transporter (ASCT2), was observed on CD19‐positive B cells in the germinal center of the lymph node (Figure 7Ab). In the metastasized lymph nodes, the close interaction of immune cells with metastatic PSTT cells was observed (Figure 7Ac), suggesting that syncytin‐1‐ASCT2 interaction is one of the candidates to mediate cell‐fusion between PSTT and immune cells.

To validate the possibility of cell fusion between PSTT and B cells, we examined the expression of CD 19 on PSTT cells in the primary lesions of the removed uterus. Although no infiltration of CD19‐positive lymphocytes was observed in most of the PSTT, in some myometrial invading lesions, CD19‐positive lymphocytes were infiltrated around LVRN‐positive PSTT cells, being in close contact with each other. Among them, LVRN/CD19 double‐positive PSTT cells were detected (Figure 7B). On the other hand, in the vascular invasion site in the uterus, abundant LVRN/CD19 double‐positive PSTT cells were sporadically observed (Figure 7C).

## 3 Discussion

PSTT has two distinctive features. First, this tumor has a semi‐allogeneic nature and then must receive sustained immune pressure from the host. Second, EVT, the origin of PSTT, has the ability to fuse with each other to become multinuclear cells in the uterus. Based on these advantages, we were able to analyze clinical samples of PSTT using a novel method for detecting DNIMA and demonstrate that exogenous genes derived from host somatic cells were incorporated into cancer cells across all chromosomes.

In contrast to somatic cell‐derived cancers, PSTT of case #1 was derived from the daughter and had allogeneic paternal antigens. Therefore, although genome mutation analysis showed no significant result and microsatellite instability was low, it must escape immune attack from the host‐maternal immune system, and the acquirement of immunotolerance‐inducing functions is critical for its survival. During the remodeling process of maternal arteries in normal pregnancy, EVT, the origin of PSTT, acquires maternal‐fetal immune tolerance from maternal immune cells [27, 28, 29]. HLA‐G1, which is specifically expressed on the cell surface of EVT, was proposed to play a main role in inducing inhibitory signals in maternal T cells or NK cells, leading to maternal‐fetal tolerance [30, 31]. However, HLA‐G1 expression was not detected either in primary or recurrent lesions of PSTT in patient #1 (Figure 1G), suggesting that PSTT had acquired an alternative system that can maintain immune tolerance. ICI therapy was temporally suppressed recurrences in consistent with recent meta‐analysis reporting that pembrolizumab was effective for high‐risk chemoresistant or relapsed PSTT [32]. On the other hand, the subsequent acute relapse in various organs after ICI therapy in patient #1 suggests the rapid increase in immunotolerant ability in PSTT during the remission period.

It is now well‐known that various carcinomas frequently produce Ig [33]. It used to be thought that only B cells produced Ig, but now there is a paradigm shift in that cancer cells themselves are known to produce Ig [34]. It has also been established that Ig can mediate effector functions that regulate various aspects of innate and adaptive immunity [35]. In addition, it was demonstrated that the modification of Ig by glycosylation leads to a marked change in Ig functions from immunoreactive to immunosuppressive roles by modulating Fc structures [36]. Recently, it was reported that sialylated cancer‐derived Ig directly interacted with effector CD4+ and CD8+ T cells through Siglecs (sialic acid‐binding Ig‐type lectins) such as SIGLEC7 and SIGLEC10 and induced the immunosuppressive effect of T cells and promotion of tumor growth, suggesting that cancer‐derived Ig itself can induce immune tolerance [37, 38]. The production of cancer‐derived Ig was also reported to be positively related to cancer metastasis and a poor prognosis in various carcinomas [27]. In this study, the distribution of CD8‐positive T cells was suppressed in the Ig‐high PSTT regions (Figure 3). Consequently, it is reasonable to assume that Ig production contributed to the initial steps of immune escape from host immune attack and cancer development in PSTT that lost HLA‐G1 expression. This finding is clinically consistent with previous reports that renal membranous glomerulopathy with granular deposition of Ig is sometimes observed as a complication of PSTT [39, 40].

By use of a new method analyzing DNIMA, the present study clearly showed that PSTT contains exogenous host maternal genes, including HLA. This incorporation of host HLA genes may lead to a decrease in the allogenic antigenicity of PSTT and contribute to inducing immune tolerance. Cell fusions can explain the mechanism of exogenous gene incorporation and subsequent transfer of donor cell‐specific functions to recipient cells [3]. In general, Ig production requires the activation of a wide range of transcription factors, including E2A, EBF, Pax5, Oct‐1 and 2, FOXO1, and FOXP1 [33, 34]. Since the exosome transfer is a partial transport system of DNAs, it is difficult for this system to achieve the whole incorporation of rearranged Ig genes into chromosomes and subsequent production of Ig. It should also be noted that transfer of the immune function from antibody‐producing cells is an established biological phenomenon that has been widely applied to the production of monoclonal antibodies by hybridoma using the cell fusion technique. In this case, gene incorporation has occurred at partial chromosome levels in HLA and ZNF regions. ZNF proteins constitute the largest transcription factor family that has finger‐like DNA binding domains and regulates multiple biological processes [41]. Since this transcription factor family was reported to play an important role in tumor development and progression [42], the incorporation of ZNF regions may be one of the critical processes that successfully transfer the donor cell‐specific functions into PSTT [43].

Mechanisms by which tumors acquire resistance to immune checkpoint inhibitors are still unknown [44]. Impaired HLA‐antigen processing and presentation were reported to induce acquired resistance in lung cancers and melanoma [45, 46]. The mutation‐associated neoantigen was also reported to be decreased in patients with acquired resistance against non‐small cell lung cancer [47]. In addition, it was demonstrated that upregulation of alternative immune checkpoint molecules such as LAG3, TIGIT, 2B4, and TIM3 is associated with adaptive resistance to PD‐1 blockade therapy [48, 49]. In this study, we showed that the expression of immunomodulating molecule, LILRB, in PSTT increased during its recurrent process (Figure 1I). In addition, we could confirm that DNIMAs in the exon region of TLR10 and those in the intron region of SIGLEC10 were progressively incorporated into PSTT, and their protein expressions also increased during the recurrent process. Furthermore, DNIMAs were detected in TLR10 mRNA in the relapsed PSTT. This result suggests that immune function‐related molecules are transferred from host immune cells to cancer cells together with their regulatory mechanisms of expression, including immunotolerance‐inducing molecules.

During placentation, human cytotrophoblast cells transform into multinucleated syncytiotrophoblast cells by cell fusion, which is induced via syncytin‐1 and ‐2, products of the two endogenous retrovirus envelope genes [26]. Syncytin‐1 was proposed to be involved in cancer cell fusion [50]. Human EVT was also reported to express syncytin‐1 [51] and is well‐known to fuse to become multinuclear cells in the uterus. PSTT expressed syncytin‐1, whereas B cells in the germinal center of lymph nodes expressed its receptor, ASCT2. Since the close interaction of immune cells with metastatic PSTT cells was observed in metastatic lymph nodes, it may be one of the prime candidate sites where cell fusion with immune cells occurs. On the other hand, the close contact of PSTT with B cells was also observed in the primary lesions infiltrated by B cells. In this site, LVRN/CD19 double‐positive PSTT cells were present. Furthermore, among vascular‐invasive PSTT cells, LVRN/CD19 double‐positive PSTT cells were abundantly observed even in the absence of surrounding B cells. These findings suggest the presence of cell fusion between PSTT and B cells in the primary lesions and the subsequent expanding process of the fused PSTT cells to the distant organs. The expressions of syncytin‐1 and 2 were also reported in other solid carcinomas such as breast, colon, prostate, endometrial cancers, and so on [3, 9]. Considering the high frequency of Ig production by various types of cancers [33, 34], it is reasonable to assume that malignant cells other than PSTT also utilize this mechanism of cell fusion with B cells to hijack the function of B cells. Since several anti‐ASCT2 therapies to inhibit amino acid transporters are currently under clinical consideration for cancer therapy [52, 53, 54], blocking the interaction with syncytin‐1 may be another promising strategy for ASCT2‐targeted therapy.

This study had several limitations. First, clinical data on temporal changes in gene profiles were only available for one PSTT patient (case #1). Since PSTT is very rare and the initial treatments were performed at different hospitals in the other three patients (cases #2, 3, and 4), genetic data for the additional cases were not available. Second, we only analyzed clinical samples in this study. To clarify the significance of cell fusion with B cells in functional changes of cancer cells, in vitro or animal experiments should be performed in the future. Third, this study provided no information on the possible involvement of extrachromosomal DNA, which is a piece of a cell's chromosome that broke off and reattached to itself to form a loop, in the development of PSTT [55]. Finally, in this study, since a single cell was recruited from a frozen section, not from an isolated or cultured single cell, the possibility of partial loss of nuclear DNA cannot be ruled out. Although this partial loss does not change the conclusion concerning the presence of exogeneous gene incorporation confirmed by of DNIMA and the absence of contamination demonstrated by Mho/Dhe‐VS, it may affect the distribution map of chromosomal polyploidy.

In conclusion, by new approaches analyzing DNIMA in gestational trophoblastic disease, this study clearly indicates that cancer cells can sequentially incorporate exogenous genes from host somatic cells. Exogenously incorporated genes included immune‐related molecules, and some of their expressions were changed step‐by‐step in accordance with recurrence. In addition, this study firstly provides the possibility that cancer cells acquire B cell‐related functions, including Ig production by exogenously acquiring host genes, probably from B cells. The mapping of DNIMA indicated that exogenous maternal genes had been incorporated at partial chromosome levels, which suggests the involvement of cell fusion in gene transfer mechanisms. These findings support the current hypothesis that the integration of exogenous genes from immune cells is a substantive mechanism for inducing immune functions favoring the survival of malignant cells, including immune resistance to immune checkpoint inhibitor therapy.

## 4 Experimental Section

### 4.1 Patients and Clinical Care

Patients were recruited from Kanazawa University Hospital and Kyoto University Hospital. Three patients were diagnosed with PSTT, two cases (patients #1 and #2) were treated at Kanazawa University Hospital, patient #3 was at Kyoto University Hospital, and patient #4 was at Kanazawa University Hospital and Chiba University Hospital. Patients' information, including laboratory data, treatments, and outcomes, was retrieved from the medical records (Table 1). This study was approved by the institutional review board of the Graduate School of Medical Sciences, Kanazawa University (2017‐041(493)), Kyoto University (R1467‐1), and Chiba University (2934). Written Informed consent for the use of tissue and blood samples in this study was obtained from all patients and their families.

### 4.2 Human Chorionic Tissues From Normal and Ectopic Pregnancy

Human chorionic tissues in the early stage of pregnancy were obtained from legal abortions of normal pregnancies (6–9 weeks of gestation, *n* = 5). Embryo‐implanting tissues were obtained from patients with ectopic pregnancy of the fallopian tube (*n* = 3). Written informed consent for the use of these tissues in this study was obtained from all donors. Analysis of these samples was approved by the Medical Ethical Committee of Kanazawa University (approval number: 2015‐009 (388)).

### 4.3 Immunohistochemistry

The archived hematoxylin‐eosin (H&E) slides were reviewed by 2 gynecological pathologists according to the recent World Health Organization classification of tumors of the female genital tract. Whole tissue sections of 4 µm were used for immunohistochemistry. Tissue localization of hCG, Mel‐CAM, LVRN, p63, SALL4, CD8, PD‐L1, PD‐L2, PD‐1, CTLA‐4, CD86, LILRB, HLA‐C, lambda light chain, CD19, CD138, TLR‐10, and Syncytin1 proteins was immunohistochemically determined by the avidin‐biotin‐peroxidase complex (VECTASTAIN ABC Kit; Vector Laboratories, Burlingame, CA, USA) method using formalin‐fixed and paraffin‐embedded sections. Primary antibodies used in the immunohistochemical study are shown in Table S1. Sections of representative blocks from the patient were deparaffinized in xylene and rehydrated in ethanol, and antigen retrieval was subsequently performed in 0.01 m citrate buffer (pH 6.0). The slides were immersed in 3% hydrogen peroxide for 10 min to block endogenous peroxidase activity and then washed with 0.05 M phosphate‐buffered saline (PBS, pH 7.4). The slides were incubated with primary antibodies overnight at 4°C in a humidified chamber. After washing, the sections were incubated for 30 min with biotin‐labeled secondary antibodies at room temperature. Next, sections were treated with the avidin‐biotin complex at room temperature. Sites of peroxidase activity were visualized by diaminobenzidine (Liquid DAB+ Substrate Chromogen System; Dako, Carpinteria, CA, USA), and the sections were then counterstained with hematoxylin and mounted.

### 4.4 Spatial Analysis of CD8‐Positive T Cells and Immunoglobulin Expression

Formalin‐fixed paraffin‐embedded tumor sections from PSTT were subjected to immunohistochemical staining for Ig light chain and CD8. Whole‐slide images were acquired and analyzed using ImageJ (NIH). Regions of interest (ROIs) were defined on consecutive sections based on Ig light chain staining intensity. The proportion of DAB‐positive tumor cells was quantified in each ROI, and regions with ≥30% positive tumor cells were classified as Ig‐high, whereas those with <30% were classified as Ig‐low. To assess spatial distribution, relative to blood vessels were identified morphologically, and ROIs were further categorized based on the distance from the nearest vessel: perivascular regions were defined as areas within 500 µm of a vessel, and distal regions as areas beyond 500 µm. CD8‐positive cells were manually counted within each ROI and normalized to area (cells/mm^2^). Comparisons of CD8‐positive cell density were performed across four groups: Ig‐high perivascular, Ig‐high distal, Ig‐low perivascular, and Ig‐low distal. To evaluate spatial organization, CD8‐positive cells were further classified as clustered or scattered. A CD8‐positive cell was defined as clustered if ≥3 neighboring CD8‐positive cells were present within a 30 µm radius. The clustered fraction was calculated as the number of clustered CD8‐positive cells divided by the total number of CD8‐positive cells within each ROI. Statistical analyses were performed using nonparametric methods. Differences among multiple groups were assessed using the Kruskal–Wallis test followed by Dunn's multiple comparison test. A *p* value < 0.05 was considered statistically significant.

### 4.5 Immunofluorescence Staining

Protein expressions of LVRN, HLA‐G1, CD19, ASCT‐2, and SIGLEC10 were confirmed by the indirect immunofluorescent antibody method. To make sections, a specimen was fixed by overnight immersion in 4% paraformaldehyde (PFA), cryoprotected by overnight immersion in sucrose‐containing PBS, and embedded in Optimal Cutting Temperature (OCT) compound (Sakura Finetek, Japan). Seven‐micrometer‐thick sections were cut using a cryostat, permeabilized with 0.5% Triton X‐100 in PBS, and incubated at 4°C overnight with primary antibodies. Primary antibodies used in the immunofluorescence staining are shown in Table S1. After being incubated at 37°C for 2 h with fluorescence‐conjugated secondary antibody and 1 µg/mL of Hoechst 33342, the sections were washed and mounted. For multiple immunofluorescence staining, primary antibodies raised in different species were used simultaneously, followed by incubation with species‐specific secondary antibodies conjugated to distinct fluorophores. The staining conditions of each antibody are listed in Table S1.

### 4.6 DNA Extraction From the Patient's Blood, Her Husband's Blood, and Her Daughter's Exfoliative Cells

DNA was prepared from 3‐mL blood samples from the patient (Maternal blood) and her husband (Paternal blood), and from exfoliative cells collected from her daughter's oral cavity (Daughter's cheek swab) using a QIAamp DNA mini kit. PBMCs were isolated by Ficoll‐Paque Plus (GE Healthcare Japan) density gradient centrifugation. DNA of FFPE tissue was extracted using NucleoSpin DNA FFPE XS (Macherey‐Nagel GmbH & Co., KG, Germany) following the manufacturer's protocol.

### 4.7 Detection of Clonal Ig Gene Recombination by Multiplex PCR

To detect V, D, and J gene segments in rearranged Igs, we performed multiplex PCR. In this PCR assay, we employed multiplex PCR primers designed for clonal Ig gene recombination in lymphoproliferation [56]. When Ig gene recombination occurs, bands appear in specific regions in each primer tube: 310∼360 bp in IgH (heavy chain) tube A, 250∼295 bp in IgH tube B, 100∼179 bp in IgH tube C, 110∼290 bp and 390∼420 bp in IgH tube D, 100∼130 bp in IgH tube E, 120∼210 bp in IgK (kappa light chain) tube A, 210∼300 bp and 350∼390 bp in IgK tube B, and 140∼165 bp in IgL (lambda light chain). All PCR reactions were performed in a final volume of 50 µL containing 10×PCR buffer I, each dNTP at 200 µm, 1 U of AmpliTaq Gold DNA polymerase (ThermoFisher Scientific, Japan), 10 ng of template DNA, and 10 pmol of primers in each multiplex PCR tube. PCR was performed under the following conditions: a single hot‐start cycle at 95°C for 10 min, followed by 40 cycles of denaturing at 95°C for 30 s, annealing at 60°C for 30 s, and extension at 72°C for 30 s. In DNA extracted from FFPE tissue, DNA degradation and fragmentation occurred, and the number of PCR cycles was increased to 45–50. Products were analyzed by 4% agarose gel electrophoresis and stained with ethidium bromide.

### 4.8 Next‐Generation Exome Sequencing‐Based Genome‐Wide Mutation Analysis

To confirm that the tumor comprised over 80% of the specimen, OCT‐embedded non‐fixed fresh frozen specimens were sectioned at 7 µm and used for hematoxylin and eosin staining. After we confirmed a sufficient amount of tumor tissue in the specimen, OCT‐embedded specimens were washed with PBS at room temperature, and DNA extraction was performed using the QIAamp DNA Mini Kit (QIAGEN, Valencia, CA, USA) following the manufacturer's protocol. Genomic DNA was analyzed by targeted massively parallel sequencing with the KAPA Hyper Plus Library Preparation Kit (Kapa Biosystems, Wilmington, MA, USA) and SeqCap EZ Human Exome Library (v.3.0) (Roche NimbleGen, Madison, WI, USA) on an Illumina HiSeq2000 sequencer. Paired‐end reads (2 × 100 bp) were aligned to the human reference genome (build hg19/GRCh37) using the Burrows‐Wheeler Aligner. Single‐nucleotide variations and insertions/deletions were identified using the Genome Analysis Toolkit (Unified Genotyper) and annotated using ANNOVAR. We first picked up the tumor‐specific mutation candidates when comparing tumor DNA with the patient's blood DNA as a control. To find actionable somatic mutation candidates, the mutation candidates were further filtered for potentially deleterious and rare (population frequency < 1% in the 1000 Genomes Project dataset) variants in 114 cancer‐associated genes [57] (referred to as NCC OncoPanel).

### 4.9 Single‐Cell Whole Genome Sequencing‐Based Genome‐Wide Mutation Analysis

To accurately pick up a single PSTT cell, OCT‐embedded non‐fixed fresh frozen specimens were sectioned at 7 µm, and we performed acetone fixation and LVRN immunohistochemical staining. The collection of individual LVRN‐positive PSTT cells was performed using the vacuum single‐cell picking device UnipicK (Nepa Gene, Chiba, Japan). To prepare the DNA library, PicoPLEX Gold Single Cell DNA‐Seq Kit (Takara, Shiga, Japan) was used following the manufacturer's protocol. Before using the extracted DNA in subsequent whole‐genome sequencing analysis, contamination of patient‐derived cells was ruled out by confirming the absence of maternal alleles at the IGLL5 locus (Figure S2B). The DNA libraries were subjected to high‐throughput sequencing with 150‐bp paired‐end reads on a DNBSEQ‐G400 sequencer (MGI Tech, Shenzhen, China). The whole genome sequencing data were analyzed using the Genome Analysis Toolkit best practices pipeline in variant discovery [58]. As controls, DNA was prepared from 3‐mL blood samples from the patient and the daughter's oral somatic cells in saliva.

### 4.10 Single‐cell Whole Genome Sequencing‐Based Copy Number Analysis

A single cell adjacent to the cell used in the single‐cell whole genome sequencing‐based genome‐wide mutation analysis was collected in the same manner using UnipicK. (Nepa Gene, Chiba, Japan). To prepare the DNA library, the KAPA Hyper Plus Library Preparation Kit (Kapa Biosystems, Wilmington, MA, USA) was used following the manufacturer's protocol. Before using the extracted DNA in subsequent sequencing analysis, contamination of patient‐derived cells was ruled out by the absence of maternal alleles at the IGLL5 locus. In order to detect the aneuploidy of the single cell, genomic DNA was analyzed by low‐coverage whole genome sequencing on an Illumina MiSeq sequencer (Illumina, San Diego, CA, USA).

### 4.11 Validation by SANGER SEQUENCING

To prepare for Sanger sequencing validation analysis, dozens of LVRN‐positive adjacent cells in the same section as single cell analysis were collected. DNA extraction and amplification were performed using GenomePlex Single Cell Whole Genome Amplification Kit (WGA4‐50RXN, Sigma‐Aldrich, Munich, Germany). Before using the extracted DNA, contamination of patient‐derived cells was ruled out by confirming the absence of maternal alleles at the IGLL5 locus (Figure 2F). All PCR reactions were performed in a final volume of 50 µL containing 10×PCR buffer I, each dNTP at 200 µm, 1 U of AmpliTaq Gold DNA polymerase (ThermoFisher Scientific, Japan), 10 ng of template DNA, and 10 pmol of primers in each PCR tube. After amplification, the resulting PCR products were purified with a MinElute Gel Extraction Kit (Qiagen) or QIAquick PCR Purification Kit (Qiagen). The purified PCR products were sequenced and detected using a DNA sequencer (3730xl DNA Analyzer; Thermo Fisher Scientific).

### 4.12 Microarray Gene Expression Analysis

Cyanine‐3 (Cy3)‐labeled cRNA was prepared from 0.2 µg of RNA using the Low Input Quick Amp Labeling Kit (Agilent Technologies) according to the manufacturer's instructions, followed by RNeasy column purification (QIAGEN, Valencia, CA, USA). Samples were hybridized to SurePrint G3 Human GE microarray 8 × 60K Ver. 3.0 (G4858A, Agilent Technologies). The slides were scanned on an Agilent DNA Microarray Scanner (G2539A) using the one‐color scan setting for 8 × 60k array slides. The scanned images were analyzed using Feature Extraction Software 11.0.1.1 (Agilent) with the default parameters (protocol AgilentG3_GX_1Color and Grid: 072363_D_F_20150612) to obtain background‐subtracted and spatially detrended processed signal intensities. The raw data have been deposited in the Gene Expression Omnibus (GEO) database under the number GSE128222R.

### 4.13 Short Tandem Repeat (STR) Polymorphism Analysis

DNA from the tumor, the patient's blood, and the patient's husband's blood was amplified with primers for 15 STR loci on 13 chromosomes using PowerPlex 16 HS System (Promega, Madison, WI, USA). PCR products were resolved by capillary electrophoresis using ABI 310 Genetic Analyzer, and genotypes were determined using GeneMapper version 4.0 software (Applied Biosystems, Warrington, UK). The genotype of the tumor tissue was compared with that of the patient's blood and her husband's blood to clarify the gestational origin of the disease.

### 4.14 Microsatellite Instability (MSI) Analysis as a Clinical Examination

Analysis was clinically performed by a hospital consignment inspection company using extracted DNA from a specimen with a tumor content of over 80%. Five nearly monomorphic mononucleotide repeat markers (BAT‐25, BAT‐26, MONO‐27, NR‐21, and NR‐24) were analyzed. MSI‐high is defined as more than two positive markers.

### 4.15 RT‐PCR Analysis

To accurately pick up dozens of LVRN‐positive cells, OCT‐embedded 4% PFA‐fixed liver metastatic lesions were sectioned at 4 µm, and we performed LVRN immunohistochemical staining. Collection of individual LVRN‐positive PSTT cells was performed using the vacuum picking device UnipicK (Nepa Gene, Chiba, Japan). Before using the extracted RNA, contamination of patient‐derived cells was ruled out by confirming the absence of maternal alleles at the IGLL5 locus. To prepare the cDNA library, Single Cell RNA Purification Kit (NORGEN BIOTEK CORP, ON, Canada) and TransPlex Complete Whole Transcriptome Amplification Kit (Sigma–Aldrich, Munich, Germany) were used following the manufacturers' protocols. For the removal of residual primers and nucleotides, a standard PCR purification kit was used, QIAamp DNA Mini Kit (QIAGEN, Hilden, Germany). The cDNA was amplified using specific primers listed in Table S2. All PCR reactions were performed in a final volume of 50 µL containing 10×PCR buffer I, each dNTP at 200 µm, 1 U of AmpliTaq Gold DNA polymerase (ThermoFisher Scientific, Japan), 10 ng of template DNA, and 10 pmol of primers in each PCR tube. After amplification, the resulting PCR products were purified with MinElute Gel Extraction Kit (Qiagen) or QIAquick PCR Purification Kit (Qiagen). The purified PCR products were sequenced and detected using a DNA sequencer (3730xl DNA Analyzer; Thermo Fisher Scientific).

## Author Contributions

KK, KA, and HF conceived the study and study design; KK, MO, YM, AHorie, and HU prepared clinical samples; KK, TK, TI, TD, SH, AHattori, SM, KH, and AT performed the experiments and data analysis; KK and HF wrote the paper; KK, MO, TD, TF, HU, KA, and HF discussed the paper; KK, MO, TF, KA, and HF contributed to funding acquisition; HF was the originator of the concept of this report. All the authors approved this paper.

## Ethics Statement

This study was approved by the institutional review board of the Graduate School of Medical Sciences, Kanazawa University (2017‐041(493)), Kyoto University (R1467‐1), and Chiba University (2934). Written Informed consent for the use of tissue and blood samples in this study was obtained from all patients and their families. Analysis of these samples was approved by the Medical Ethical Committee of Kanazawa University (approval number: 2015‐009 (388)).

## Conflicts of Interest

The authors declare no conflicts of interest.

## Supporting information




**Supporting File**: advs76071‐sup‐0001‐SuppMat.docx

## Data Availability

The data that support the findings of this study are available in the supplementary material of this article.
