# Supplementary material for: Placental Site Trophoblastic Tumor Acquires Immune Functions by Incorporating Host Maternal Genes
Source: Adv Sci (Weinh). 2026 Jun 23;13(50):e76071. doi: 10.1002/advs.76071 (PMC13336403; doi:10.1002/advs.76071)
Supplement: Supplementary file 1 [file ADVS-13-e76071-s001.docx]

**Supporting information**

**Advanced Science**

**Placental site trophoblastic tumor acquires immune functions by incorporating host maternal genes**

Kyosuke Kagami^1*^, Masanori Ono^1,2,3^, Yasunari Mizumoto^1,4^, Tatsuhito Kanda^1^, Takashi Iizuka^1^, Takiko Daikoku^5^, Shin-ichi Horike^6^, Akira Hattori^7^, Akihito Horie^8,9^, Sachiko Minamiguchi^10^, Tomoko Fujiwara^11^, Kazuyoshi Hosomichi^12,13^, Atsushi Tajima^13^, Hirokazu Usui^14^, Kaoru Abiko^1*^, and Hiroshi Fujiwara^1,15,16*^

**Supporting information list**

**•** Supporting Table S1 and S2

**•** Supporting Figures S1-S4

**Table S1 Antibodies (staining condition)**

| **REAGENT or RESOURCE** | **SOURCE** | **IDENTIFIER** |
| --- | --- | --- |
| Anti-LVRN, clone 5-23 (1:100) | Fujiwara, H et al. [1] | N/A |
| Anti-LVRN, polyclonal (1:500) | Maruyama, M et al.[2] | N/A |
| Anti-hCG (1:50) | Roche | Cat#760-2650 |
| Anti-Mel-CAM (1:100) | Abcam | Cat#ab75769, RRID:AB_2143375 |
| Anti-p63 (1:100) | Abcam | Cat#ab735, RRID:AB_305870 |
| Anti-PD-L1 (1:250) | Abcam | Cat#ab213524, RRID:AB_2857903 |
| Anti-PD1 (1:250) | Abcam | Cat#ab52587, RRID:AB_881954 |
| Anti-CD86 (1:100) | Abcam | Cat#ab53004, RRID:AB_869050 |
| Anti-HLA-C (1:100) | Abcam | Cat#ab193432, |
| Anti-HLA-G1 (1:500) | Abcam | Cat#ab7758, RRID:AB_306052 |
| Anti-human IgG Lambda Light Chain (1:250) | Abcam | Cat#ab124719, RRID:AB_10974949 |
| Anti-CD19 (1:300) | Abcam | Cat#ab134114, RRID:AB_2801636 |
| Anti-TLR10 (1:100) | Abcam | Cat#ab53631, RRID:AB_883039 |
| Anti-SLC1A5/ASCT2 (1:1000) | Abcam | Cat#ab237704 |
| Anti-SALL4 (1:100) | Abnova | Cat#H00057167-M03, RRID:AB_566160 |
| Anti-PD-L2 (1:100) | Merck | Cat#MABC1120 |
| Anti-CD8 (1:100) | Agilent | Cat#M710301-2 |
| Anti-CTLA-4 (1:100) | Santa　Cruz Biotechnology | Cat#sc-376016, RRID:AB_10988256 |
| Anti-CD138/ Syndecan-1 (1:250) | Santa　Cruz Biotechnology | Cat#sc-12765, RRID:AB_626900 |
| Anti-SIGLEC10 (1:500) | Sigma-Aldrich | Cat#HPA027093, RRID:AB_1856858 |
| Anti-Syncytin1 (1:100) | Bioss | Cat#bs-2962R, RRID:AB_11106881 |
| Anti-ILT (1:100) | Santa Cruz | Cat# sc-515288 |

[1]	H. Fujiwara, T. Higuchi, S. Yamada, T. Hirano, Y. Sato, Y. Nishioka, S. Yoshioka, K. Tatsumi, M. Ueda, M. Maeda, S. Fujii, *Biochem Biophys Res Commun* **2004**, *313* (4), 962.

[2]	M. Maruyama, A. Hattori, Y. Goto, M. Ueda, M. Maeda, H. Fujiwara, M. Tsujimoto, *J Biol Chem* **2007**, *282* (28), 20088. https://doi.org/10.1074/jbc.M702650200.

**Table S2 Primers for detecting variants using gDNA and cDNA**

***Primers for detecting variants using gDNA***

Human IGLL5 Forward (5’- ACAACACCCCGGTATTCTGTC -3)

Human IGLL5 Reverse (5’- CTGTAGGGGCCACTGTCTTC-3’)

Human IGLL5 (semi-nested) Forward (5’- ACAACACCCCGGTATTCTGTC -3)

Human IGLL5 (semi-nested) Reverse (5’- GCTGTAGCTTCTGTGGGACTT-3’)

Human IGL Forward (5’- CCACCCCATGGTGTTACCAA -3)

Human IGL Reverse (5’- CACCTAGGACGGTCAGCTTG-3’)

Human TLR10 Forward (5’- GTCAAGTCTGCGGGAACCTT -3)

Human TLR10 Reverse (5’- TCAACAACGGAGACATGGCA-3’)

Human TLR10 (semi-nested) Forward (5’- GCATCACCCTCTGCTGTCAT -3)

Human TLR10 (semi-nested) Reverse (5’- GGTTGGAACCTTACTCCAACCT-3’)

Human SIGLEC10 Forward (5’- AGTACTGGGAGAGACGTGCC-3)

Human SIGLEC10 Reverse (5’- CAGGGAAATGTCCCATACCTGGAAG-3’)

Human SIGLEC10 (nested) Forward (5’- GGCTTCCCTCTGGGTAAAGG -3)

Human SIGLEC10 (nested) Reverse (5’- GAGTCCTCTCCTCGTCCCAT-3’)

HLA-DQA2 Forward (5’- AGCCCAACACCCTCATTTGT -3)

HLA-DQA2 Reverse (5’- ACAACGCCCTCCCACTTTC -3’)

HLA-DQA2 (nested) Forward (5’- ACTCTGCATTCTGACCTCAACA -3)

HLA-DQA2 (nested) Reverse (5’- TGTCTGGAAGCACCAACTGAA -3’)

***Primers for detecting variants using cDNA***

Human TLR10 Forward (5’- ACTTTTGGGCAAGCACCTGA -3)

Human TLR10 Reverse (5’- GCACAAATGCCACACATGCT-3’)

Human TLR10 (semi-nested) Forward (5’- TCCAAGTGTTCCAAGGGTGT -3)

Human TLR10 (semi-nested) Reverse (5’- GCACAAATGCCACACATGCT-3’)

Human GAPDH Forward (5’- GAGAAGGCTGGGGCTCATTT -3)

Human GAPDH Reverse (5’- AGTGATGGCATGGACTGTGG -3’)

Human GAPDH (nested) Forward (5’- GGGAGCCAAAAGGGTCATCA -3)

Human GAPDH (nested) Reverse (5’- GATGGCATGGACTGTGGTCA -3’)

**Supporting Figure S1. Dissection of PSTT cells**

Manual dissection of CD19- and CD138-free PSTT regions in the primary lesion of PSTT. Scale bars: 200 μm.

**Supporting Figure S2. Evaluation of contamination by LOH detection**

(A) Metastatic LVRN-positive PSTT cells at the final stage before and after single-cell picking (white arrowheads). Scale bars: 100 μm. (B) LOH region on the IGLL5 gene locus. This site showed only paternal SNVs, indicating that there was no contamination of the patient's own cells.

**Supporting Figure S3. Highly repeat-rich regions defined as the top 5% retrotransposon contents**

The relationship between DNIMA-positive sites and transposon hotspots such as LINE, SINE, LTR retrotransposons, ERV, and SVA retrotransposon in the PSTT genomes were shown in the chromosomal mapping. The occupancy rate of the above retrotransposons for each 1 Mb section from the whole genome data was calculated and the highest density regions as the top 5% were defined as a region of dense retrotransposon. There was no significant relationship between DNIMA-positive sites and transposon hotspots.

**Supporting Figure S4. AT-rich sequence sites in PSTT**

Highly AT-rich genomic regions defined as the top 5% AT content were mapped in the whole chromosomes of PSTT. The map showed no significant relationship between DNIMA-positive sites and AT-rich regions.
